# Overlapping functions of *Cdx1*, *Cdx2*, and *Cdx4* in the development of the amphibian *Xenopus tropicalis*

**DOI:** 10.1002/dvdy.21901

**Published:** 2009-04

**Authors:** Laura Faas, Harry V Isaacs

**Affiliations:** Department of Biology, University of York, YorkUnited Kingdom

**Keywords:** Cdx, *Xenopus*, Hox, caudal, mesoderm, endoderm, morphogenesis, wnt

## Abstract

Using *Xenopus tropicalis*, we present the first analysis of the developmental effects that result from knocking down the function of the three Cdx genes present in the typical vertebrate genome. Knockdowns of individual Cdx genes lead to a similar range of posterior defects; compound Cdx knockdowns result in increasingly severe posterior truncations, accompanied by posterior shifts and reduction of 5′ Hox gene expression. We provide evidence that Cdx and Wnt3A genes are components of a positive feedback loop operating in the posterior axis. We show that Cdx function is required during later, but not early stages of development, for correct regional specification of the endoderm and morphogenesis of the gut. Our results support the hypothesis that during amphibian development the overall landscape of Cdx activity in the embryo is more important than the specific function of individual Cdx proteins. *Developmental Dynamics 238:835–852, 2009*. © 2009 Wiley-Liss, Inc.

## INTRODUCTION

The Cdx family of homedomain transcription factors has conserved functions in the development of several animal groups. The prototype of the Cdx family is the *caudal* gene, which is required for normal posterior development of *Drosophila* (MacDonald and Struhl, 1986; Mlodzik and Gehring, 1987; Mlodzik et al., 1987). The Cdx class, together with the Gsx and Pdx classes, comprise the ParaHox family of homeobox genes. In the cephalochordate amphioxus, the single representatives of each ParaHox class are clustered, having a similar genomic organization to that of the closely related Hox gene family (Brooke et al., 1998). In the vertebrate lineage sequential genome duplication has led to cluster expansion and subsequent degeneration, such that the typical vertebrate genome contains the remains of four ParaHox gene clusters but only three Cdx genes (*Cdx1*, *Cdx2*, and *Cdx4*). Only *Cdx2* remains clustered with members of the Pdx and Gsx classes (Ferrier et al., 2005; Illes et al., 2009).

Analysis of Cdx gene expression in several vertebrate species, including mouse, chick, fish, and frog shows that Cdx genes are initially expressed during gastrula stages in overlapping domains in the mesoderm; subsequently, they are expressed in all three germ layers in dynamic patterns in the posterior of the developing body axis (Gamer and Wright, 1993; Meyer and Gruss, 1993; Marom et al., 1997; Pillemer et al., 1998; Reece-Hoyes et al., 2002; Gaunt et al., 2003; Lohnes, 2003). The expression patterns of the Cdx genes resemble that of the Hox genes, in that all Cdx genes are typically expressed in the posterior of the embryo but each individual Cdx gene exhibits a different anterior boundary of expression. This produces a nested set of Cdx gene expression, establishing a gradient of Cdx activity along the posterior axis (Marom et al., 1997; Pillemer et al., 1998).

Gene inhibition and overexpression studies in several chordates including ascidians, fish, frogs, chick, and mouse have shown that the role of Cdx genes in posterior development has been conserved during evolution (Subramanian et al., 1995; Pownall et al., 1996; Epstein et al., 1997; Katsuyama et al., 1999; Bel-Vialar et al., 2002; van den Akker et al., 2002; Davidson et al., 2003; Lohnes, 2003; Chawengsaksophak et al., 2004; Shimizu et al., 2005; van Nes et al., 2006). Cumulative evidence suggests that *Cdx* factors act as transducers of positional information by regulating the boundaries of *Hox* gene expression domains (Charite et al., 1998; van den Akker et al., 2002; Gaunt et al., 2004,^2008^).

In keeping with their regulatory role during axis development, Cdx proteins are crucial factors involved in anteroposterior patterning of the digestive tract. The regulation of gene expression in the digestive tract by Cdx proteins in vertebrates is well documented (Beck, 2004; Guo et al., 2004). Furthermore, *Cdx2* misexpression has been implicated in homeotic anterior to posterior transformation in the gut epithelium (Mutoh et al., 2002; Silberg et al., 2002), indicating a role for Cdx genes in establishing regional identity.

Single and double gene knockout and knockdown experiments in various species support roles for Cdx gene function in anteroposterior patterning of the main body axis. However, establishing a clear picture of the overall role of the Cdx family in these processes is difficult because individual Cdx genes exhibit significant overlap of expression during early development. It is, therefore, likely that there is some functional redundancy among the Cdx genes and that some degree of compensation may occur in the event of single and double gene deficiencies. This underlines the importance of undertaking studies involving inhibition of the activity of the full complement of Cdx genes present in model vertebrate organisms.

In *Xenopus* the three Cdx genes were originally designated *Xcad1*, *Xcad2*, and *Xcad3* (Pillemer et al., 1998). However, for the sake of consistency, we have adopted the human and mouse nomenclature for the frog Cdx genes. Thus, *Xcad1* is the orthologue of amniote *Cdx2*, and *Xcad2*, and *Xcad3* are the orthologues of *Cdx1* and *Cdx4*, respectively.

In the present study, we have undertaken a systematic analysis of the developmental effects resulting from single and compound knockdown of the three *Cdx* family members in the frog *Xenopus tropicalis* using translation blocking antisense morpholino oligos (MOs). Our study is the first to present data on the developmental effects that result from knocking down the activity of the three *Cdx* genes present in the typical vertebrate genome.

Data in this investigation show that compound knockdown of the three *Xenopus Cdx* genes gives rise to a highly penetrant, severe truncation of the posterior axis. Similar ranges of developmental abnormalities are seen when each individual *Cdx* gene is knocked down, indicating that the *Cdx* genes have overlapping functions in posterior axial development. However, the increased severity of the phenotypes in the compound knockdowns argue in favor of a cooperative effect of *Cdx* genes on posterior patterning.

We present data indicating that the amphibian Cdx genes are components of gene regulatory pathways, involving Wnt ligands and 5′ Hox genes, that are required for morphogenesis and patterning in the posterior of the main body axis during postgastrula stages. In addition, we find a requirement for Cdx function in the normal morphogenesis and regional specification of the amphibian gut during later stages of development. Data in this study support the hypothesis that, during early amphibian development, individual *Cdx* genes have overlapping function and that in a given region of the embryo, it is the overall level of Cdx activity that is relevant, rather than the specific function of individual Cdx proteins.

## RESULTS

### Antisense Morpholino Oligos That Block *Cdx* Translation

**Fig. 1:**
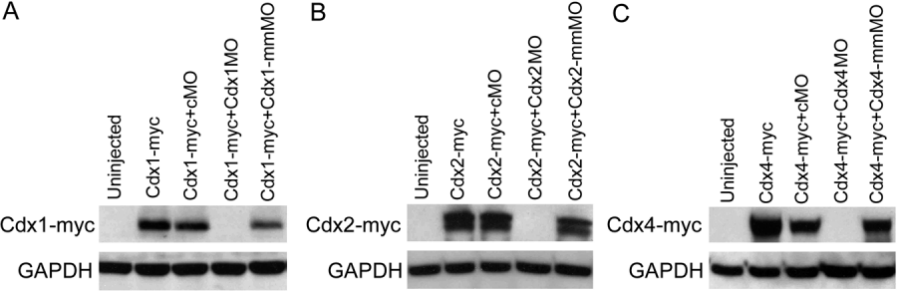
Inhibition of Cdx protein translation by morpholino oligos (MOs). **A–C:** Western blots showing that the Cdx1, Cdx2, and Cdx2 MOs but not the standard control MO or the corresponding five base mismatch control MOs block the translation of myc-epitope tagged Cdx target proteins in embryos. Early cleavage stage embryos were injected with 10 pg of Cdx-myc mRNA or co-injected with 10 pg of Cdx-myc mRNA+ 10 ng of standard control morpholino (cMO) or 10 ng of the corresponding translation blocking MOs (Cdx1 MO, Cdx2 MO, or Cdx4) or 10 ng of the corresponding five mismatch MOs (Cdx1-mmMO, Cdx2-mmMO, or Cdx4-mmMO). Data are presented for the set-1 morpholinos.

Antisense morpholino oligos (AMOs) targeted to the initiating AUG and/or the 5′ UTR of the *Xenopus tropicalis* *Cdx1*, *Cdx2*, and *Cdx4* mRNAs were tested for their ability to block translation of myc-epitope tagged Cdx proteins (Fig. 1). A standard control MO (cMO) has little effect on the efficiency of Cdx protein translation in whole embryos. In contrast, the Cdx1, Cdx2, and Cdx4 MOs (set-1 in the Experimental Procedures section) efficiently block translation from the corresponding target mRNA, whereas MOs with sequences differing from the translation blocking MOs by five bases (mmMOs) have relatively little effect on translation from the target mRNAs. A second set of translation blocking Cdx MOs (set-2 in the Experimental Procedures section) also inhibit translation of their respective targets but less efficiently (data not shown). Unless stated otherwise the set-1 Cdx morpholinos were used in all subsequent experiments.

### Analysis of the *Cdx* Knockdown Phenotype

**Fig. 2:**
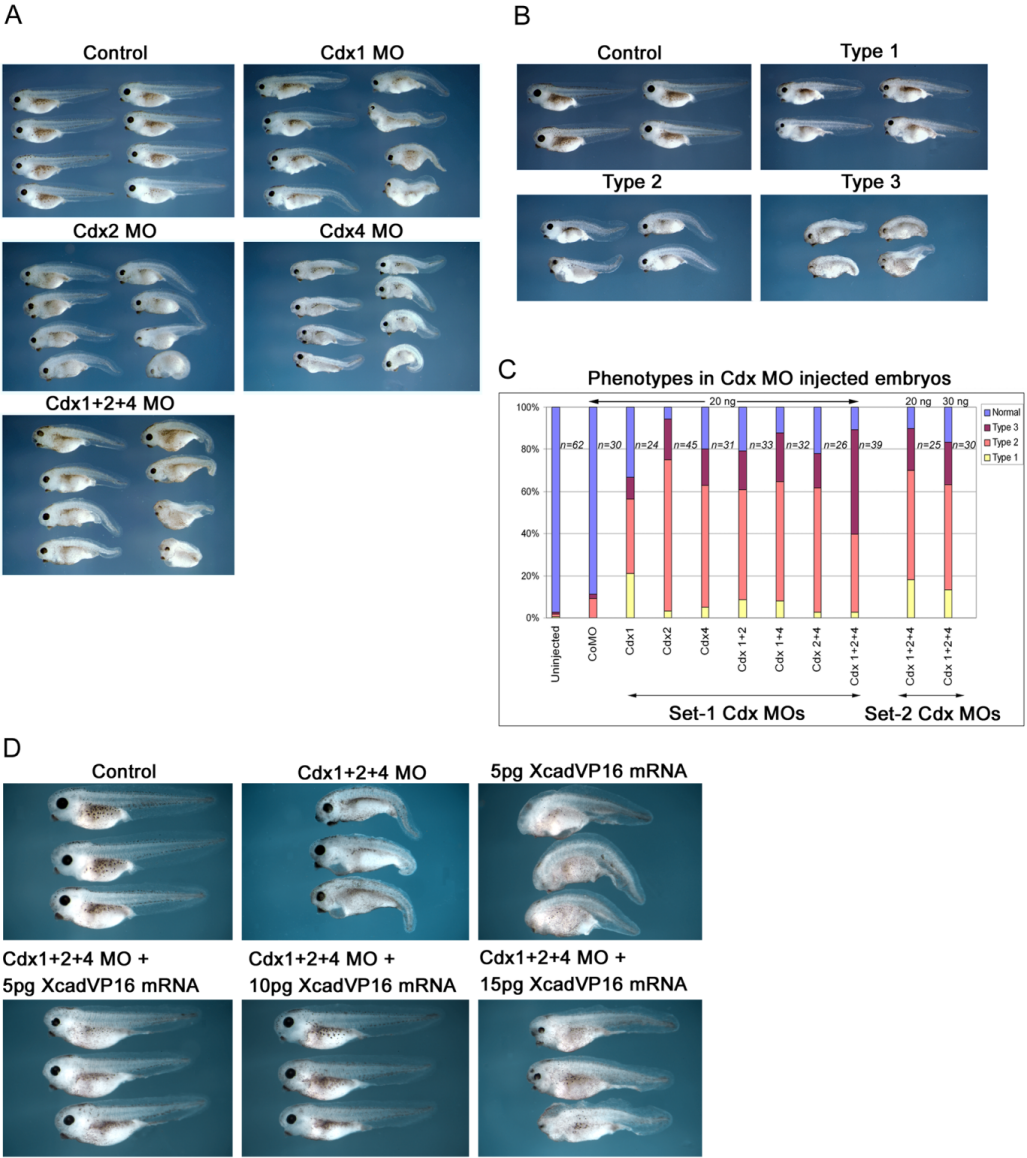
The phenotype of Cdx knockdown embryos. **A:** Single and triple knockdowns of Cdx1, Cdx2, and Cdx4 give rise to a similar range of phenotypes. Single knockdown embryos were injected with 20 ng each of Cdx1-A morpholino oligo (MO), Cdx2-A MO, or Cdx4-A MOs. Triple knockdown embryos were injected with a 20 ng total of the set-1 Cdx1, Cdx2, Cdx4 MOs. **B:** The phenotypes of larval stage 41 control embryos and embryos exhibiting type 1, 2, and 3 axial defects produced by the knockdown of Cdx function. **C:** A bar chart showing the proportions of type1, 2, and 3 axial defect embryos in control uninjected embryos, standard control morpholino injected embryos and embryos injected with combined or single set-1 Cdx or set-2 Cdx translation blocking morpholinos. In each case, the total mass injected is made up of equal quantities of each contributing MO. Total injected quantities and n values for each group are indicated on the chart. **D:** XcadVP16 mRNA rescues the compound Cdx1, Cdx2, and Cdx4 knockdown phenotype. Embryos were injected with a 20 ng of total of the Cdx1, Cdx2, and Cdx4 MO, together with the indicated quantities of XcadVP16 mRNA and cultured until larval stage 41.

Injection of the set-1 Cdx1, Cdx2, and Cdx4 MOs, individually or collectively, in the range of 10 ng to 20 ng/embryo, results in highly penetrant effects on posterior axial development (Fig. 2A–C, and data not shown). In contrast developmental abnormalities occur at low frequency in uninjected embryos or embryos injected with the standard control MO (5% and 10%, respectively, in a typical experiment). Consistently, the triple knockdown using the second set of Cdx MOs (set-2), results in a similar range of posterior abnormalities (Fig. 2D, and data not shown).

Injection of more than 20 ng of the triple Cdx knockdown MO combination results in embryos with highly truncated posterior structures by tail bud stages (Supp. Fig. S1, which is available online). However, high concentrations of the Cdx MOs results in reduced survival of embryos into later tail bud stages, accompanied by degeneration of the endodermal mass.

Figure 2A shows the phenotypes of control embryos and embryos injected with Cdx MOs at larval stage 41. Of interest, all single or compound knockdown embryos showed a similar spectrum of posterior truncations. In each case the total amount of MO injected is the same, with equal contribution from each constituent MO. To facilitate comparison between experiments, we have classified the range of knockdown phenotypes at swimming larval stage 41 into types 1, 2, and 3 (Fig. 2B). Type 1 embryos display a mild shortening of the axis and a “pigeon chested” appearance; development of the head is relatively normal. Type 2 embryos exhibit a shortened, curved, or kinky axis and mild anterior defects, including foreshortened head and reduction of the eye size. Type 3 embryos show a very short, curved axis, severe reduction of tail outgrowth, and anterior defects.

Figure 2C shows the proportions of type 1, 2, and 3 phenotypes at larval stage 41 in control uninjected embryos, control MO injected embryos and embryos injected with the Cdx MOs used in this study. As mentioned earlier, single and double Cdx knockdowns using the set-1 MOs result in similar profiles of phenotypes; however, the triple Cdx knockdown results in a much higher proportion of the more severe type 3 phenotype. Triple Cdx knockdown using the set-2 MOs produced similar effects on axial development but with reduced number of type 3 embryos (Fig. 2C), consistent with their reduced efficiency at blocking target Cdx protein translation (Fig. 1, and data not shown).

### Specificity of the *Cdx* Knockdown Phenotype

As an important test of the specificity of knockdown MOs, we show that the mutant Cdx protein Xcad-VP16 rescues the effects on axial development resulting from Cdx knockdown. XcadVP16 consists of an amino-terminal fusion of the transcriptional activation domain from the viral VP16 protein to the DNA binding domain of *Xenopus laevis* Cdx4 protein (Isaacs et al., 1998). Xcad-VP16 mimics the activity of native Cdx protein but the 5′ end of the Xcad-VP16 mRNA is not targeted by the Cdx translation blocking MOs.

Figure 2D shows that injection of 5 pg of XcadVP16 mRNA produces an anterior truncation phenotype, similar to that previously reported for overexpression of *Xenopus laevis* Cdx4 (Isaacs et al., 1998). Injection of XcadVP16 mRNA in the 10- to 15-pg range produces a very strong phenotype characterized by a vestigial dorsal axis (data not shown). Injecting a combination of Cdx1, Cdx2, and Cdx4 MOs results in the typical, highly penetrant posterior truncation phenotype (100%, n = 32). However, co-injection of Xcad-VP16 mRNA, in the 5- to 10-pg range, with the Cdx MOs rescues axial abnormalities in a dose dependent manner (85%, n = 72). The phenotype of embryos co-injected with the Cdx MOs and 15 pg of XcadVP16 mRNA exhibit mild anterior truncations, similar to that produced by the injection of low doses of XcadVP16 mRNA alone.

Of interest, this anterior truncation phenotype is mild compared with that resulting from injection of 15 pg of XcadVP16 mRNA alone (data not shown). These data indicate that the observed effects in this rescue experiment result from integration of Cdx activity present within the embryo and suggest that the overall level of Cdx activity plays a critical role in regulating axial development.

As another test of the specificity of the knockdown effects, five base mismatch MOs were injected into embryos. At a given concentration, five mismatch MOs give far lower numbers of abnormal embryos than do the translation blocking MOs. Co-injecting a combination Cdx1, Cdx2, and Cdx4 MOs results in 67% (n = 87) abnormal embryos, compared to 5% abnormal for uninjected stage control embryos (n = 137). In contrast, co-injecting the same amounts of the corresponding mismatch MOs results in just 12% (n = 128) abnormally developing embryos. This is similar to the approximately 10% of abnormal embryos typically observed after injection of the standard control MO (Fig. 2C, and data not shown).

### Tissue Organization in *Cdx* Knockdown Embryos

Our data indicate that injection of the triple Cdx knockdown MO combination in the range 20 to 40 ng per embryo produces a highly penetrant phenotype. Based upon these findings, in subsequent experiments, we used injection of these amounts or pro rata amounts for injection into individual cells. Due to the endodermal degeneration observed in tail bud embryos injected with 40 ng of the triple Cdx knockdown MO cocktail, injections in experiments requiring culture to later tail bud stages were limited to 20 ng. To control for nonspecific effects that might arise from the injection of a given mass of MO, in experiments comparing the effects of single, double, or triple knockdowns, the total amount of MO injected was divided equally between the constituent MOs.

**Fig. 3:**
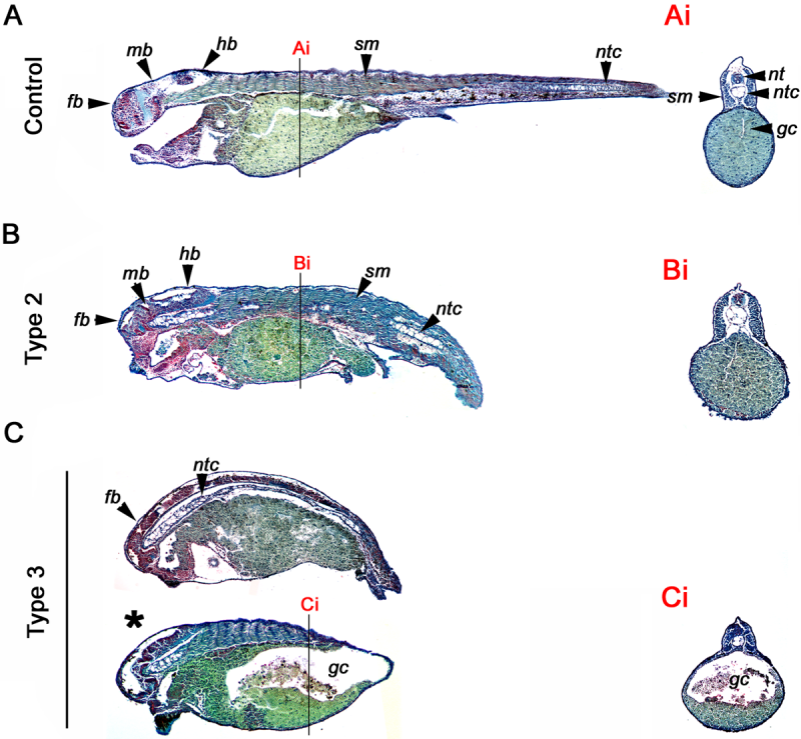
Histology of Cdx phenotype embryos. Despite shortening of the body axis the main axial and paraxial tissues are present. **A–C:** Sagittal sections of control, type 2, and type 3 embryos at larval stage 41, respectively. The insets Ai, Bi, and Ci are transverse sections at the indicated axial level. Note the disorganized structure of the anterior central nervous system and enlarged gut cavity in Type 3 embryos. fb, forebrain; gc, gut cavity; hb, hindbrain; mb, midbrain; nt, neural tube; ntc, notochord; sm, somite. Red asterisk indicates enlarged brain ventricle.

We undertook histology on type 2 and type 3 embryos to analyze the effects resulting from triple Cdx knockdown on the differentiation and patterning of the major tissues. The length of the main body axis is severely reduced in the knockdown embryos; despite this, within the remaining vestigial axis the organization of the major axial tissues (notochord and neural tube) is relatively normal (Fig. 3). Paraxial tissues, such as the somites, are present and exhibit segmentation similar to that seen in controls (Fig. 3A–C). However, we note that the typical three ventricle organization of forebrain, midbrain, and hindbrain of the anterior nervous system is lost and is reduced to a single enlarged ventricle in the high grade type 3 embryos (Fig. 3C). Another striking feature apparent in type 3 embryos is gross enlargement of the gut cavity relative to control embryos (Fig. 3 Ci).

### Overlapping *Cdx* Function

There is good evidence that the overlapping expression and activity of Cdx proteins means that some redundancy in function exists between Cdx family members (Davidson and Zon, 2006; van den Akker et al., 2002). We investigated this by undertaking the phenotypic rescue of double knockdown embryos by the third nontarget Cdx protein.

**Fig. 4:**
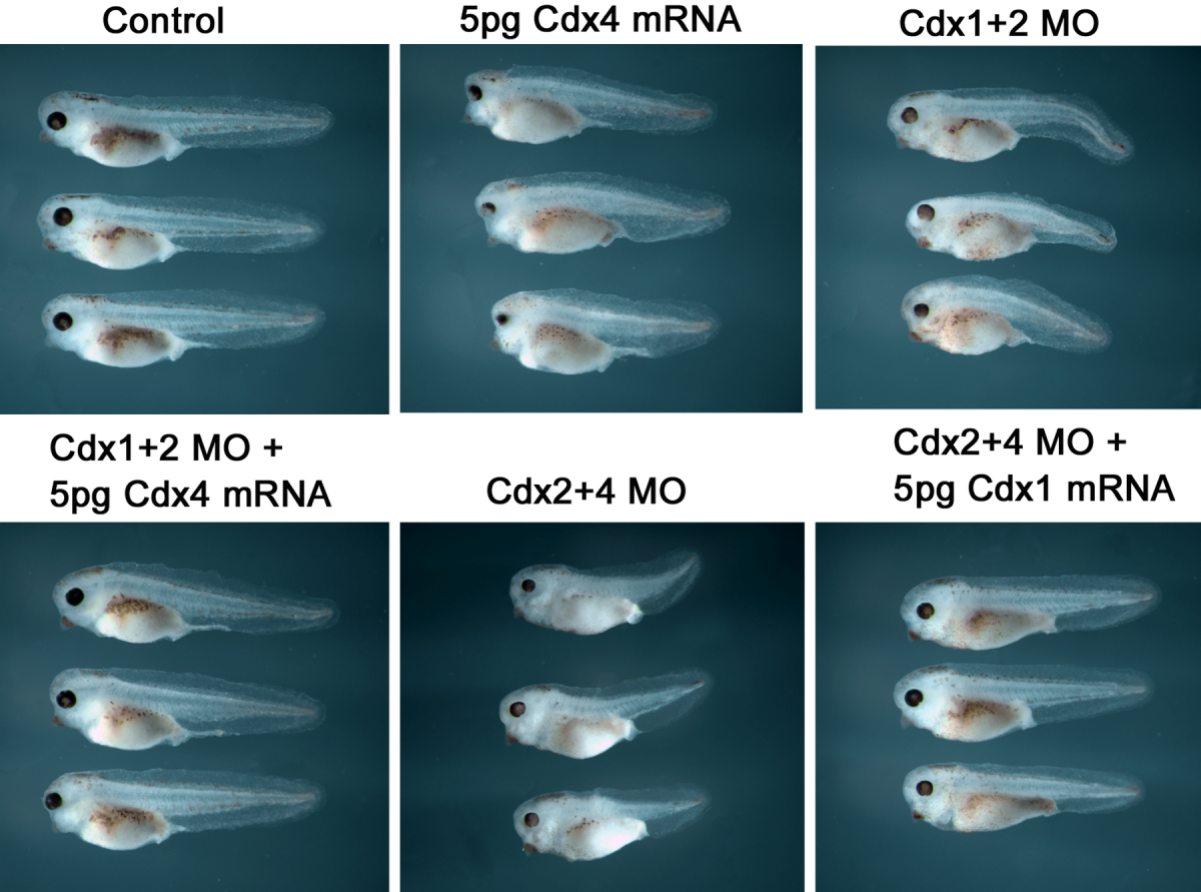
Overlapping Cdx function. The phenotype of double Cdx knockdown embryos at larval stage 41 can be rescued with a third nontargeted Cdx protein, indicating overlapping function between amphibian Cdx family members. Cdx1+2 knockdown embryos (20 ng of MO total dose) were rescued with the indicated amounts of *Cdx4* mRNA. Cdx2+4 double knockdown embryos (20 ng of MO total dose) were rescued with *Cdx1* mRNA.

Figure 4 shows the typical anterior truncation phenotype caused by overexpressing *X. laevis* Cdx4 (Isaacs et al., 1998). Rescue of the Cdx1+2 MO phenotype with *X. laevis* *Cdx4* mRNA increases the number of normal embryos from 25% (n = 29) to 43% (n = 28). Similar effects were observed in the double Cdx2+4 knockdown rescued with *X. laevis Cdx1* mRNA. Injection of Cdx2 and Cdx4 MOs results in a mild but penetrant effect on posterior axial development. However, co-injection of *Cdx1* mRNA increases the number of normal embryos from 5% (n = 37) to 40% (n = 40). These experiments demonstrate that raising the level of a single Cdx protein can compensate for knockdown in the function of two other Cdx genes.

### *Cdx* Regulation of Hox Gene Expression

Functional studies in several vertebrate models, including chick, mouse, and fish, indicate that Hox genes are targets of Cdx regulation and interference with normal Hox gene expression, in part, accounts for the observed derangement of posterior development resulting from Cdx inhibition (Subramanian et al., 1995; Bel-Vialar et al., 2002; van den Akker et al., 2002; Davidson et al., 2003).

**Fig. 5:**
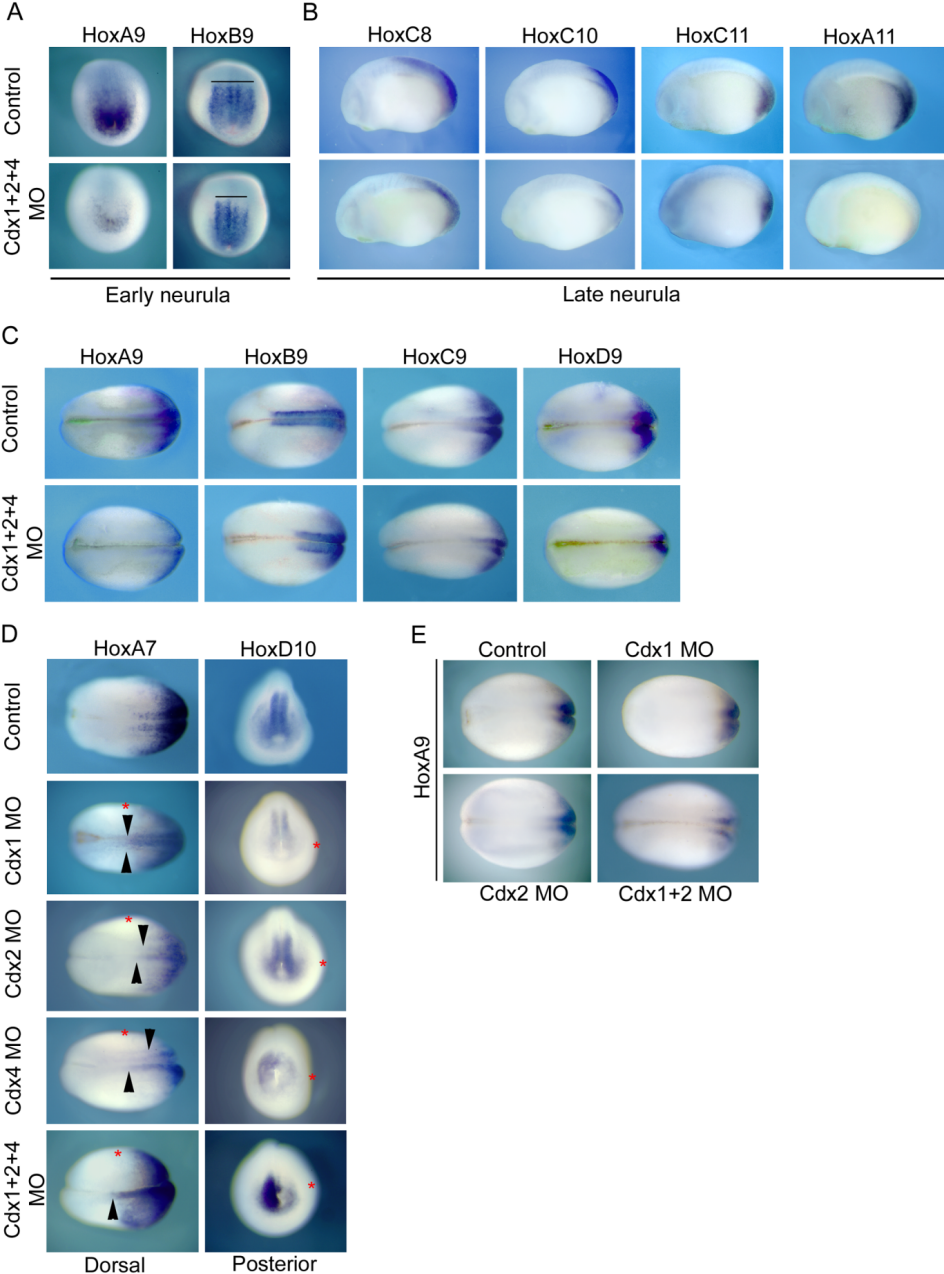
Effects of Cdx knockdown on Hox gene expression. Cdx function is required for the normal expression of multiple posterior Hox genes. A–E: Whole-mount in situ hybridizations of the indicated Hox genes. A: Late gastrula stage 13 embryos, and B–E show late neurula stage 20 embryos. A–C: Embryos were injected bilaterally with 20 ng of Cdx1, Cdx2, and Cdx4 MOs. **A:** Panels with *HoxA9* shows posterior views with dorsal to the top. Panels with *HoxB9* show dorsal views with anterior to the top. Black bars indicate lateral extent of expression domain in neural tube. **B:** Lateral views with anterior to the left. **C:** Dorsal views, anterior to the left. **D:** Representative control embryos and embryos injected unilaterally with 10 ng of Cdx1, Cdx2, Cdx4 MOs, or the triple Cdx MO combination; injected side is indicated with a red asterisk (left panels are dorsal views with anterior to the left, and right panels are posterior views with anterior to the top. **E:** A control uninjected embryo and embryos injected bilaterally with 20 ng of Cdx1, Cdx2, or the Cdx1 and Cdx2 combination (dorsal views with anterior to the left). Black arrows indicate anterior boundary of expression within the neural tube on injected and contralateral uninjected sides.

Previous studies in *Xenopus laevis* support a role for Cdx regulation of Hox genes. Cdx overexpression up-regulates the expression of posterior Hox genes (paralogue groups 6 to 9) and results in anterior shifts in limits of expression along the main body axis (Pownall et al., 1996; Epstein et al., 1997; Isaacs et al., 1998). Complementary experiments using antisense RNA mediated *Cdx1* knockdown or overexpression of an antimorphic Cdx4 protein result in reduced posterior Hox gene expression (Epstein et al., 1997). However, the effects on Hox expression resulting from compound Cdx inhibition, as well as the role of each Cdx protein, remains unclear. The present study addresses this issue by examining the effects on the expression of several posterior Hox genes (paralogue groups 7 to 11) in single and compound Cdx knockdown embryos (Fig. 5).

### Effects of *Cdx* Knockdown on the Initial Expression of Posterior Hox Genes

The onset of expression from paralogue group 7 and 9 Hox genes commences in the late gastrula in the blastopore region and posterior neural plate, regions that overlap with Cdx expression (Pillemer et al., 1998; Reece-Hoyes et al, 2002). Preliminary experiments indicated that standard control MO injections have no effect on Hox gene expression (data not shown). In contrast, bilateral, compound knockdown of Cdx1, Cdx2, and Cdx4 greatly down-regulates *HoxA9* expression levels, as well as its lateral and anterior boundaries in both the neural plate and posterior mesoderm at the late gastrula stage (Fig. 5A). Similarly, the size of the *HoxB9* expression domain in the neural plate is reduced, in Cdx triple-knockdown embryos (Fig. 5A). In the case of *HoxB9*, the most obvious change is a reduction in the lateral extent of its expression boundary. These data indicate that the normal expression of posterior Hox genes in late gastrula/early neurula stage embryos requires Cdx function.

### Effects of *Cdx* Knockdown on Hox Expression in the Late Neurula

At late neurula stages both Cdx and posterior Hox genes are expressed with distinct anterior boundaries within the trunk region of the main body axis in both the mesoderm and the neural tube (Godsave et al., 1994; Pillemer et al., 1998; Reece-Hoyes et al., 2002). The nested expression patterns of HoxC genes from paralogue groups 8, 10, and 11 are illustrated in Figure 5B, where *HoxC8* has the most anterior and *HoxC11* the most posterior boundary of expression.

Analysis of posterior Hox gene expression at the late neurula stage reveals that bilateral, compound knockdown of Cdx1, 2, and 4 leads to down-regulation in the expression levels and/or a posterior shift in the boundaries of expression in the neural tube and mesoderm. For example, distinct posterior shifts in expression boundaries in the posterior mesoderm and neural tube are apparent with *HoxC8*, *HoxC10*, and *HoxC11* in Cdx knockdown embryos (Fig. 5B). In the case of *HoxA11*, both the expression levels and the anterior boundary of expression in mesoderm and neural tube are dramatically affected by compound Cdx knockdown (Fig. 5B).

### *Cdx* Inhibition and the Four Hox Gene Clusters

To determine whether Cdx function is required for the expression of paralogous genes in the four different Hox clusters we examined the effects of Cdx inhibition on the expression of paralogue group 9 genes from the HoxA, HoxB, HoxC, and HoxD clusters. Figure 5C shows that Cdx1+2+4 knockdown causes an overall reduction in the expression domains of all four Hox9 paralogue genes. The posterior shift of *HoxA9*, *HoxC9*, and *HoxD9* expression boundaries is evident in the neural tube and mesoderm. In contrast to the changes observed in the late gastrula (Fig. 5A), we do not see reduction in the lateral extent of *HoxB9* expression in the neural tube at this stage. However, as with *HoxA9*, *HoxC9*, and *HoxD9*, there is a posterior shift in the *HoxB9* neural tube expression boundary.

### Hox Gene Expression in Single and Double *Cdx* Knockdown Embryos

Having seen that triple Cdx knockdown alters 5′ Hox gene expression, we were interested to compare the effects on Hox expression resulting from single and double Cdx knockdowns. Expression of *HoxA7* and *HoxD10*, which preliminary data indicated are strongly affected by Cdx inhibition, were analyzed in embryos injected unilaterally with Cdx MOs. For this type of analysis, unilateral injections were performed to facilitate visualization of boundary shifts resulting from Cdx knockdown relative to the control uninjected contralateral side.

Figure 5D shows that triple Cdx knockdown markedly reduces *HoxA7* expression in the posterior neural tube and results in a posterior shift in the boundary of *HoxD10* in the neural tube. Cdx knockdown also leads to down-regulation and posterior shifts in the expression boundaries of *HoxA7* and *HoxD10* within the posterior mesoderm.

We find that knockdown of individual Cdx genes has less effect on the expression of these genes than does the triple knockdown (Fig. 5D). Unilateral Cdx4 knockdown has the strongest effects, causing a posterior shift of *HoxA7* expression in the neural tube and inhibition of *HoxD10* in the posterior mesoderm, relative to the uninjected contralateral side. Cdx2 knockdown results in a slight posterior shift of *HoxA7* in the neural tube but little effect on *HoxD10* expression, whereas Cdx1 knockdown has little effect on the expression of either *HoxA7* or *HoxD10* expression. We note that the severity of the effect resulting from individual Cdx knockdown is related to the anterior boundary of expression for that gene. Thus knockdown of *Cdx4*, which has the most anterior boundary of expression, has the most effect on Hox expression, whereas *Cdx1* has the most posterior boundary of expression and its knockdown has least effect on Hox expression.

As is the case with *HoxD10*, individual knockdown of either Cdx1 or Cdx2 has little effect on *HoxA9* expression (Fig. 5E). However, the double Cdx1 and Cdx2 knockdown leads to some down regulation of *HoxA9* expression in the posterior neural tube (Fig. 5E), but less dramatically than the triple Cdx knockdown (Fig. 5A,C). Taken together, these data suggest that the overall level of Cdx gene function is critical for posterior Hox gene expression.

### Analysis of Gastrulation in *Cdx* Compound Knockdown Embryos

Our data and previous studies indicate widespread effects on posterior Hox gene expression in response to Cdx gene inhibition. However, at present it is unclear whether the observed posterior truncation phenotypes resulting from Cdx inhibition in vertebrate embryos can be attributed to effects on Hox expression or indicates an independent role for Cdx function in posterior morphogenesis (for discussion, see van den Akker et al., 2002). Given that *Xenopus* Cdx genes are expressed within the mesoderm during gastrulation, it is possible that they might be involved in regulating germ layer specification and gastrulation movements (Pillemer et al., 1998; Reece-Hoyes et al., 2002).

**Fig. 6:**
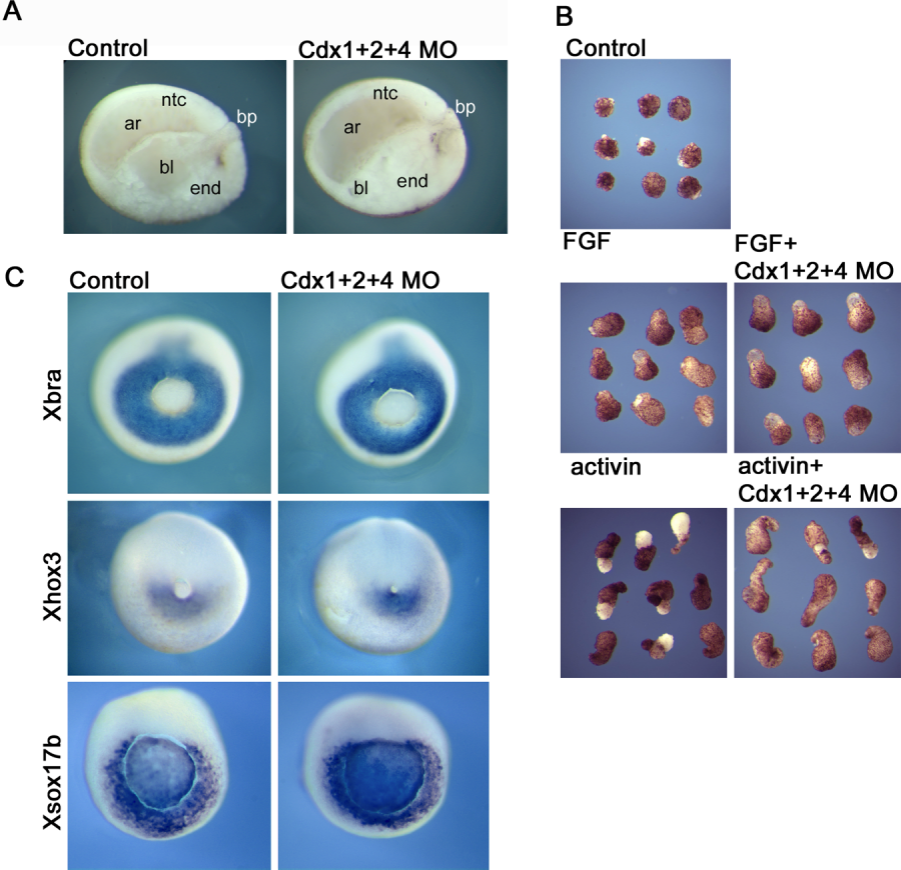
Effect of Cdx knockdown during gastrulation. **A:** The major morphogentic movements of gastrulation are not affected in Cdx knockdown embryos. Sagittal sections are shown of a control embryo and an embryo injected with a total dose of 40 ng of the triple Cdx1+2+4 MO combination at late gastrula stage 13 (anterior to the left, dorsal to the top). ar, archaenteron; ntc, prospective notochord; blc, blastocoel; bp, blastopore; end, endoderm. **B:** Growth factors induce elongation in animal cap explants from Cdx knockdown embryos. Animal hemisphere explants were taken at mid-blastula stage 8 from uninjected embryos and embryos injected with 40 ng of Cdx1, Cdx2, and Cdx4 MOs and cultured to neurula stage 18. Growth factor treatment was with 10 U/ml recombinant FGF4 or 50 U/ml recombinant activin-A (act). **C:** The expression of early germ layer markers is unaffected in Cdx knockdown embryos. Expression is shown of the endodermal marker *Xsox17b* and the mesodermal markers *Xhox3* and *brachyury* (*Xbra*) in triple Cdx knockdown embryos (40-ng total dose) and controls (posterior views, dorsal to the top). *Xbra*, *Xhox3*, and *Xsox17b* embryos are at gastrula stages 11.5, 12.5, and 11, respectively.

We analyzed late gastrula knockdown embryos to investigate whether the axial defects observed in Cdx knockdowns are related to defects in movements of the germ layers during gastrulation. Figure 6A shows that the anterior extent of the archenteron is similar in control and Cdx knockdown embryos after closure of the blastopore at the end of gastrulation. This indicates that, at the end of gastrula stages, no major abnormalities in the cell movements that drive involution and elongation of dorsal axial tissues are apparent. In the specimens presented, the blastocoel is somewhat smaller in the knockdown embryo, which perhaps suggests that morphogenetic activity is not completely normal in Cdx knockdown embryos. However, we note that, even in control embryos, the timing of blastocoel displacement and the size of the archenteron varies considerably at the end of gastrulation.

The typical convergent extension movements that occur during the elongation of axial mesoderm in gastrula and neurula stages can be mimicked by animal cap explants treated with mesoderm inducing growth factors such activin and fibroblast growth factors (FGFs).

We investigated whether Cdx knockdown interferes with growth factor-induced convergent extension movements (Fig. 6 B). Untreated animal cap explants show the typical rounded, nonelongated appearance at late neurula stage 18. Treatment with activin or FGF leads to elongation of the animal caps explants. However, MO-mediated knockdown of Cdx function in the animal caps does not inhibit the mild elongation induced by FGF or the more dramatic elongation behavior induced by activin treatment. We note that the extrusion of yolky cells from one end of the highly polarized structures induced by activin treatment is reduced by Cdx knockdown suggesting that there are some effects on morphogenetic activity. However, our data indicate that inhibition of Cdx activity does not have major effects on the convergent extension movements that drive the elongation of dorsal mesodermal tissues during gastrula and neurula stages.

### Germ Layer Specification in *Cdx* Knockdown Embryos

We then investigated the effect of Cdx knockdown on the specification of the germ layers by analyzing the expression several germ layer markers. The T-box gene *brachyury* is a key regulator of mesoderm specification in vertebrates (Showell et al., 2004). The eve-related homeobox gene *Xhox3* is expressed in ventrolateral mesoderm and has been implicated in the specification of ventroposterior mesoderm in *Xenopus* (Ruiz i Altaba and Melton, 1989). The HMG-box transcription factor *Sox17b* is a key regulator of endoderm specification (Hudson et al., 1997). Figure 6C shows that Cdx knockdown does not interfere with the early expression of these genes in gastrula stage embryos and, therefore, inhibition of Cdx function does not block the initial specification of the mesoderm or endoderm.

It is important to note that our analysis has focused on gene expression and cell movements in the mid-to-late gastrula phase and we cannot exclude the possibility of effects on cell movements and gene expression during earlier gastrula stages following Cdx knockdown.

### Axial and Paraxial Mesoderm in Neurula Stage *Cdx* Knockdown Embryos

**Fig. 7:**
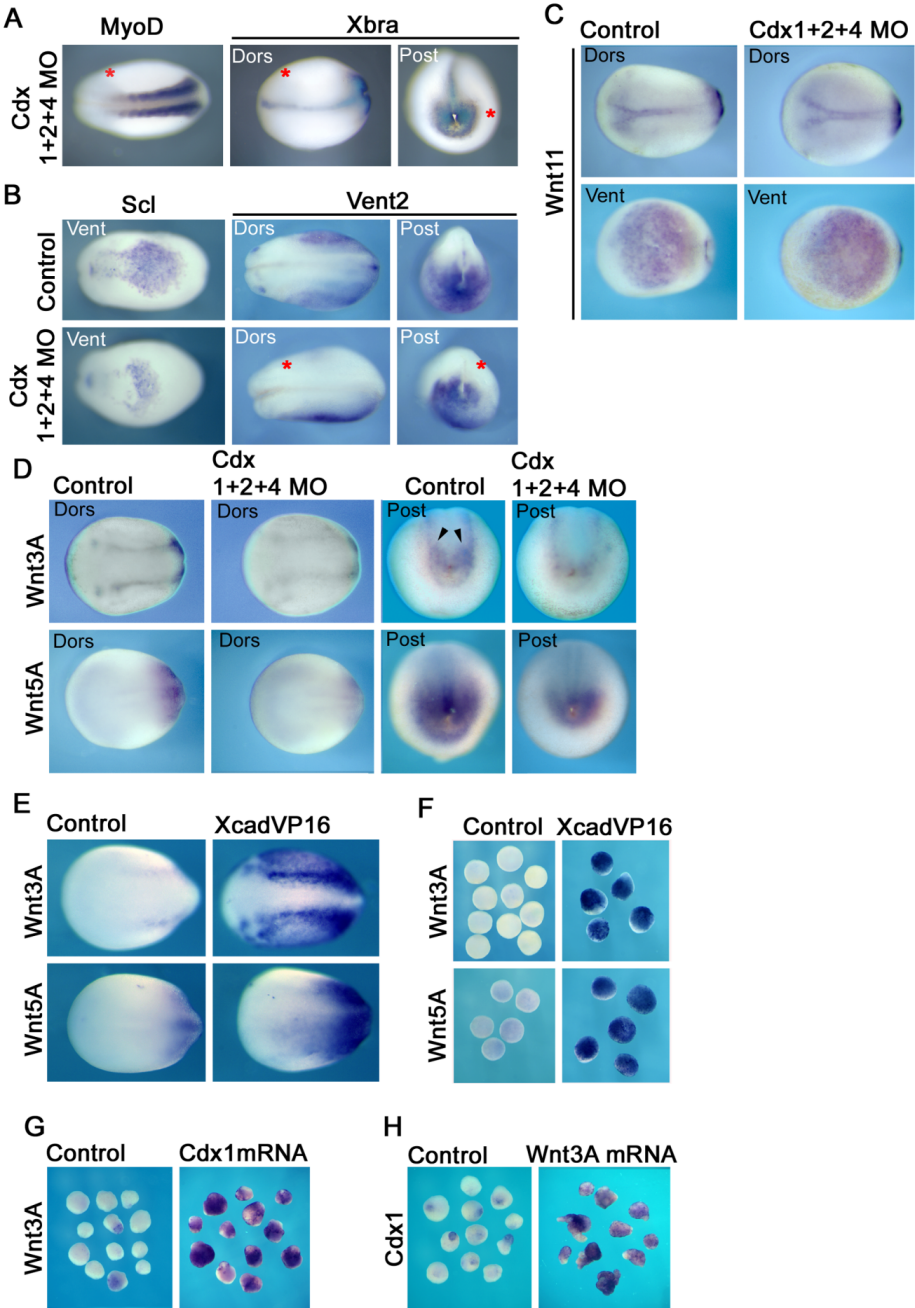
Effects of Cdx knockdown during post-gastrula stages. All samples are *Xenopus tropicalis* except (F–H) which are *Xenopus laevis* explants, which were used because their bigger size allowed easier of processing for in situ hybridization. **A:** Expression of the trunk/posterior mesodermal markers *MyoD* and *brachyury* (*Xbra*) is unaffected in Cdx knockdown embryos at late neurula stage 20. Embryos were injected unilaterally with 10 ng of combined Cdx1, Cdx2, and Cdx4 morpholino oligos (MOs); red asterisk indicates injected side. Left and middle panels are dorsal views with anterior to the left; right panel is a posterior view with dorsal to the top. **B:** Expression of the ventral mesodermal markers *Scl* and *Vent2* is down-regulated in Cdx MO knockdown embryos at stage 20. Embryos were injected bilaterally (*Scl*) or unilaterally (*Vent2*) with 10 ng per blastomere of the triple Cdx MO combination at the two-cell stage. Left panels are ventral views with anterior to the left; middle panels are dorsal views with anterior to the left and right panels are posterior views with dorsal to the top. Red asterisk indicates the injected side. **C:** *Wnt11* expression is unaffected in triple Cdx knockdown embryos (40-ng total dose) at the early neurula stage 14. Top panels are dorsal views with anterior to the left, and bottom panels are ventral views with anterior to the left. **D:** Posterior expression of *Wnt5A* and *Wnt3A* is down-regulated in triple Cdx knockdown embryos (40-ng total dose) at early neurula stage 14. Dorsal and posterior views are indicated. Dorsal views with anterior to the left and posterior views with dorsal to the top. Black arrows indicate posterior paraxial mesoderm. **E:** Increasing Cdx activity in embryos up-regulates *Wnt3A* and *Wn5A* expression. Embryos were injected with15 pg Xcad-VP16 mRNA. Dorsal views anterior to the left. **F:** Increasing Cdx function in animal caps up-regulates *Wnt3A* and *Wnt5A* expression. Animal cap explants from embryos injected with 50 pg of Xcad-VP16 mRNA were cultured until early neurula stage 14. **G:** *Wnt3A* expression is upregulated in animal caps from embryos injected with 250 pg of *Cdx1* mRNA. (H) *Cdx1* expression is up-regulated in animal caps from embryos injected with 50 pg of *Wnt3A* mRNA.

As we have seen a major aspect of the Cdx knockdown phenotype is the failure of posterior axis extension, together with the loss of posterior tissue types, including paraxial (somites) and axial mesoderm (notochord). We have investigated the early specification of these tissues types in postgastrula stages, using *brachyury* as a marker of posterior and axial mesoderm during neurula stages and *MyoD* as a marker of paraxial mesoderm. Figure 7A shows that in embryos unilaterally injected with the Cdx1, Cdx2, and Cdx4 MO combination expression of *MyoD* and *brachyury* is unaffected relative to the contralateral uninjected side, indicating that the reduced posterior axial extension of the posterior mesoderm is not simply due to inhibition of paraxial and axial mesoderm formation during neurula stages. Expression of markers of ventral and ventrolateral mesoderm was also examined in Cdx knockdown embryos (Fig. 7 B). *Scl*, which codes for a basic helix–loop–helix (bHLH) transcription factor, is a marker of the haemangioblast lineage in the ventral mesoderm (Mead et al., 1998). *Vent2* codes for a homeoprotein and is a marker of ventroposterior mesoderm (Onichtchouk et al., 1998). We find that Cdx inhibition results in a considerable reduction of both markers. *Scl* expression is down-regulated in the region giving rise to the ventrally located blood islands, and *Vent2* expression is also reduced in the lateral and posterior mesoderm around the closed blastopore by Cdx knockdown.

### *Cdx* Regulation of Wnt Gene Expression

Canonical Wnt signaling by ligands such as Wnt3A play key roles in specification and patterning the vertebrate posterior axis (Takada et al., 1994). Noncanonical Wnt signaling is also required for axial development; ligands such as Wnt11 and Wnt5A are involved in regulating axial morphogenesis through the planar cell polarity signaling pathway (Wallingford, 2004). Therefore, interference with canonical or noncanonical Wnt signaling pathways provides a conceivable mechanism that might underlie the derangement of posterior axial development that results from Cdx inhibition.

Our results show that the expression domains of *Wnt11* in the dorsal midline, ventral region, or posterior axis remain unaffected (Fig. 7 C) in early neurula stage embryos. In contrast, normal *Wnt3A* expression in the posterior paraxial mesoderm is markedly down-regulated in Cdx knockdown embryos and the size of the *Wnt5A* domain is also considerably reduced (Fig. 7 D). Conversely, increasing Cdx activity by overexpression of XcadVP16 massively up-regulates the expression of both *Wnt3A* and *Wnt5A* in both whole embryos and animal cap explants relative to controls (Figures 7E and F, respectively).

It has been reported that Wnt3A regulates the expression of both *Cdx1* and *Cdx4* during zebrafish and mouse development (Ikeya and Takada, 2001; Prinos et al., 2001; Shimizu et al., 2005; Pilon et al., 2006). Our data show that increasing Cdx activity in the embryo up-regulates *Wnt3A* expression, raising the possibility that *Wnt3A* and Cdx genes are components of a positive feedback loop operating during the development of the posterior axis. Further support for this hypothesis is provided by Figure 7G,H, which shows that Cdx1 overexpression up-regulates *Wnt3A* expression and overexpression of Wnt3A up-regulates *Cdx1* expression in animal cap explants.

### *Cdx* Knockdown Inhibits Ventral and Dorsal Elongation in the Neurula to Tail Bud Period

**Fig. 8:**
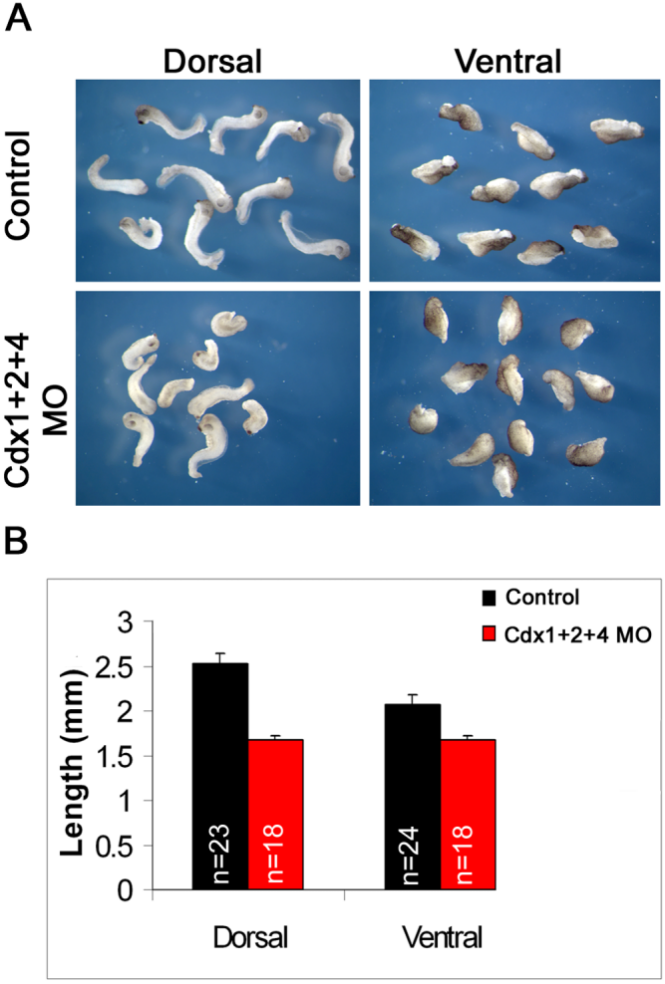
Effects of Cdx knockdown on neurula stage explants. **A:** Cdx knockdown inhibits the elongation of dorsal and ventral explants at control stage 27. Explants were taken at neurula stage 16 from uninjected embryos and embryos injected with a total 40 ng of combined Cdx1, Cdx2, and Cdx4 morpholino oligos (MOs). **B:** A bar chart showing the average length of dorsal and ventral explants of Cdx knockdown and control embryos at control stage 28 (mean ± SE; n values per group are indicated on the chart).

As previously discussed, the severe axial truncations resulting from Cdx knockdown are unlikely to result from effects on morphogenetic activity during gastrula stages. During gastrula and neurula stages, the dorsal axial tissues are the main drivers of morphogenetic movements in the amphibian embryo. However, it has been reported that cell re-arrangements in ventral tissues also contribute to axial elongation in postneurula embryos (Larkin and Danilchik, 1999). We investigated the effect of Cdx knockdown on the elongation of dorsal and ventral explants prepared at mid-neurula stage 16 and then cultured to early tail bud stage 27. We find that Cdx knockdown significantly inhibits post-neurula elongation in both dorsal and ventral explants (two experiments, dorsal *P* < 0.0001 and ventral *P* < 0.0001; Fig. 8A,B). Elongation of these explants is a distinct phenomenon from tail bud extension, which does not commence until after stage 27 (Larkin and Danilchik, 1999). It is important to note that tail bud growth is also abnormal in Cdx knockdown embryos; thus, the axial reduction that characterizes the Cdx knockdown phenotype results from effects on both morphogenesis in the neurula to tail bud stage, and subsequent tail bud outgrowth.

### Effects of *Cdx* Knockdown on Endoderm Gene Expression

The development of the endoderm derived structures is clearly abnormal in Cdx knockdown embryos. However, the expression of the early endodermal marker *Sox17b* is unaltered in gastrula stage Cdx knockdown embryos, suggesting that the observed abnormalities are not due to deficient endoderm specification. Therefore, we investigated the effects of Cdx knockdown on endodermal gene expression during later development. To discriminate the effect of Cdx knockdown on endoderm development from dorsal axial patterning, Cdx knockdown MOs were targeted to the presumptive endoderm by injecting into vegetal pole region.

**Fig. 9:**
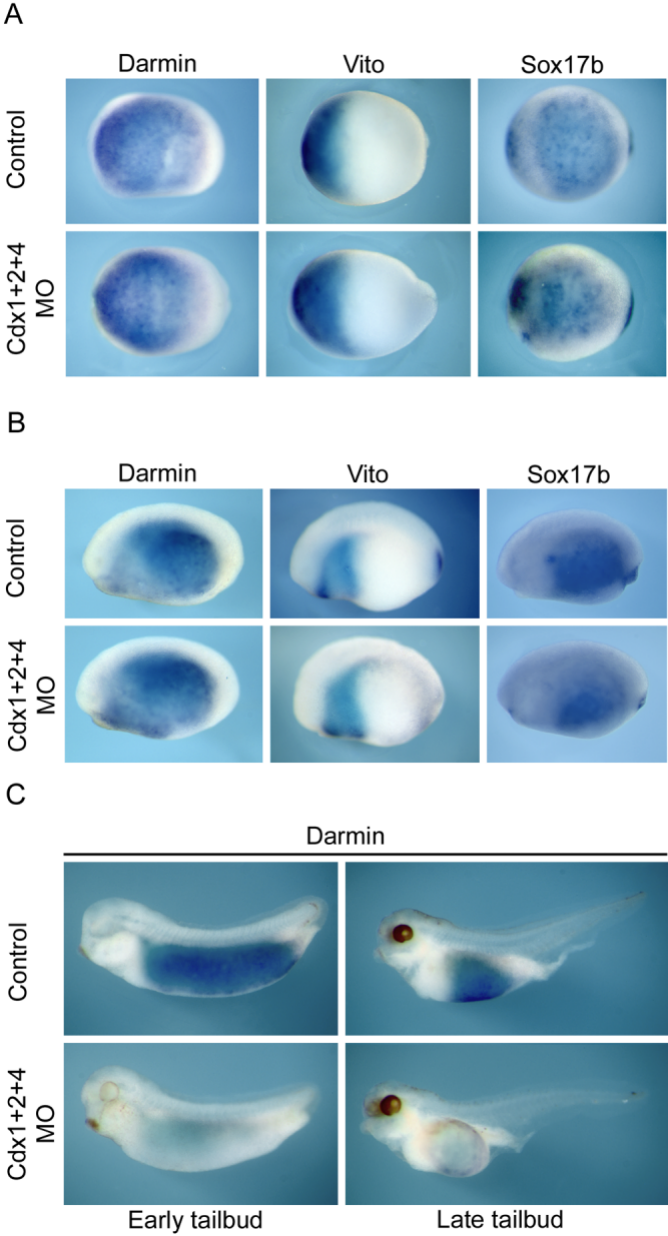
Effect of Cdx knockdown on endoderm development. **A–C:** Whole-mount in situ hybridizations to the indicated endodermal markers from neurula to tadpole stages. **A,B:** Show that expression of the endodermal marker genes *Darmin*, *Vito*, and *Sox17b* is unaffected in (A) early neurula stage 14 embryos (ventral views, anterior to the left and (B) late neurula stage 20 embryos (lateral view, anterior to the left) injected with a total of 20 ng of Cdx1, Cdx2 and Cdx4 MOs into the four vegetal hemisphere cells at the eight cell stage. **C:** *Darmin* expression is down-regulated in Cdx knockdown embryos at later stages of development. Lateral views of tail bud stage 32 (left panels) and tadpole stage 42 (right panels) embryos (anterior to the left) are shown.

The expression of *darmin*, *vito*, and *Xsox17b* was analyzed at different neurula and tail bud stages. *Vito* encodes a sodium solute transporter protein expressed in the ventral midgut at tail bud stages (Costa et al., 2003), whereas *darmin/endocut* encodes a secreted metalloproteinase expressed in the endoderm and early midgut (Costa et al., 2003; Pera et al., 2003). At early neurula stages, the expression of these endodermal markers was not affected in triple *Cdx* knockdown embryos (Fig. 9 A). The expression patterns of all three markers are also little affected at late neurula stages (Fig. 9 B). Together, our findings show that expression of the early regulator of endoderm specification *Xsox17b* is unaffected in Cdx knockdown embryos from early gastrula through late neurula stages, and that regional markers, such as *darmin* and *vito*, are also unaffected, indicating that the early endoderm is specified and regionalized. In contrast, by late tail bud/larval stages the expression of *darmin* is down-regulated indicating that at these later stages the development of the endoderm is abnormal (Fig. 9 C). These findings are in keeping with the reported Cdx expression in the gut; *Cdx1* and *Cdx2* are not detected at high levels in the posterior endoderm until tail bud stages (Pillemer et al., 1998; Chalmers et al., 2000; Reece-Hoyes et al., 2002).

**Fig. 10:**
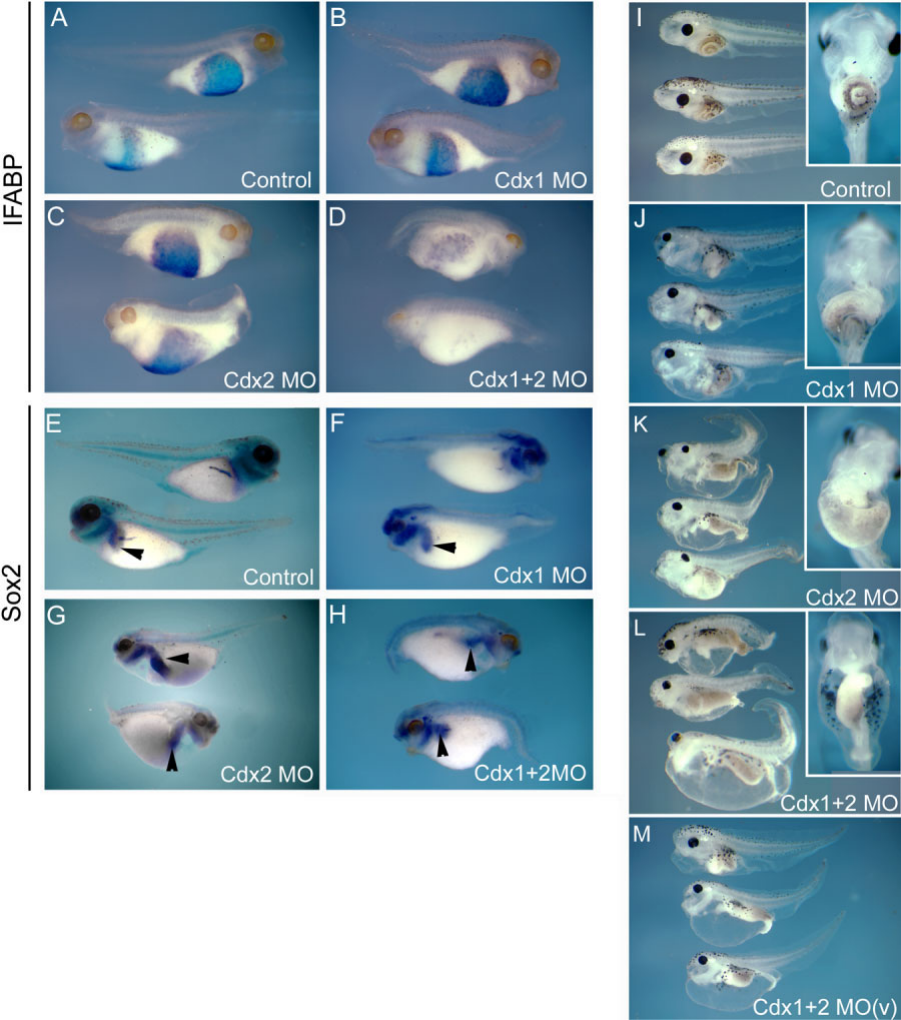
Effects of Cdx knockdown on gut development. **A–H:** Knockdown of Cdx1 and Cdx2 results in down-regulation of the posterior endodermal marker *Intestinal Fatty Acid Binding Protein* (*IFABP*). Exp (E–H). Expression of the anterior gut marker Sox2 is unaffected by Cdx1 and Cdx2 knockdown. Both right lateral and left lateral views are presented of larval stage 41 embryos injected with 20 ng of either Cdx1 morpholino oligo (MO), Cdx2 MO, or their combination. Black arrows indicate anterior gut. **I–M:** Knockdown of Cdx1 or Cdx2 results in abnormal gut morphogenesis. Embryos shown are at larval stage 45 and were injected with 20 ng of total of either Cdx1 MO, Cdx2 MO, or their combination. Main images are lateral views with anterior to the left. Insets are ventral views of the gut region showing details of gut coiling. M: Shows lateral view of embryos injected into the vegetal cells at the eight-cell stage with 10 ng of total of combined Cdx1 and Cdx2 MOs. Note that targeting the MOs to the presumptive endoderm results in embryos with abnormal gut morphology but normal development of the dorsal axis.

Both *Cdx1* and *Cdx2* have been implicated in regulating regional identity along the anteroposterior axis of the developing gut (Guo et al., 2004). To address the role of these Cdx genes in regional specification of the amphibian gut during later development, we analyzed the effects of single and compound Cdx1 and Cdx2 knockdowns on the expression of two gut regional markers, *intestinal fatty acid binding protein* (*IFABP*) and *Sox2*. *IFABP* is expressed in the small intestine, whereas *Sox2* is expressed in a region of the anterior gut including the esophagus and stomach (Chalmers et al., 2000). Injection of control MO has no effect on IFABP or Sox2 expression. Figure 10A–H shows *IFABP* and *Sox2* expression patterns in control and Cdx knockdown, larval stage 41 embryos. As normal coiling of the gut results in distinct, asymmetric gut morphology, including displacement of the stomach to the left side of the embryo both left and right lateral views of embryos are shown. Both single and compound knockdowns of Cdx1 and Cdx2 result in clear axial shortening and, even as early as larval stage 41, effects on gut coiling are apparent (compare the appearance of the anterior gut in Figure 10E vs. 10G). The expression of IFABP is not affected in either of the single knockdowns, despite obvious effects on posterior axial extension (Fig. 10B,C). However, combined Cdx1+Cdx2 knockdown results in a dramatic down-regulation of *IFABP* expression throughout its normal expression domain (Fig. 10 D). In contrast, either single or compound Cdx1+Cdx2 knockdowns have little effect on the expression of *Sox2* (Fig. 10E–H).

### Gut Morphology in *Cdx1* and *Cdx2* Knockdown Larval Stage Embryos

To analyze the effect of Cdx1/Cdx2 knockdown on the morphology of the late differentiated gut, embryos were injected with *Cdx1*, *Cdx2*, or *Cdx1* and *Cdx2* MOs and the resulting phenotypes were analyzed at larval stage 45. At this stage, the larval gut exhibits complex, stereotypical coiling (Chalmers and Slack, 2000; Fig. 10I and insert). Gut coiling was markedly disrupted in both single Cdx1 or Cdx2 knockdown embryos, resulting in a much simpler, U-shaped or S-shaped structure (65%, n = 23 and 78%, n = 23, respectively; Fig. 10J,K and inserts). Double Cdx1+2 knockdowns produced a more severe phenotype, with the majority of the embryos displaying an almost linear, tubular gut structure (66%, n = 30; Figure 10L and insert).

It could be argued that such effects on gut development might be secondary to the primary effects on axial development. As a way to discriminate the effect of Cdx knockdown in gut development from axial patterning, combined Cdx1 and Cdx2 knockdown MOs were targeted to the presumptive endoderm of the vegetal hemisphere. Figure 10M shows that embryos injected with Cdx1+Cdx2 MOs into the four vegetal hemisphere cells at the eight cell stage exhibit rather normal axial development. However, severe disruption of gut coiling is still observed, with embryos either exhibiting a simple, linear, or an S-shaped gut (51% and 20%, respectively, n = 45).

## DISCUSSION

The presence of multiple Cdx genes in vertebrate genomes with overlapping expression and upstream regulators raises questions with regard to redundancy in Cdx gene function (Lohnes, 2003; Keenan et al., 2006). This issue is addressed in the present study, where for the first time we report the developmental effects of knocking down the function all three Cdx genes in a vertebrate genome. In common with many gene knockdown studies, we are unable to determine the amount of residual Cdx activity present in knockdown embryos and the degree to which this remaining activity ameliorates the observed effects. Despite this caveat, several conclusions can be drawn from our study.

### Overlapping *Cdx* Function During Axial Elongation

Our data show that single knockdowns of amphibian Cdx1, Cdx2, or Cdx4 result in a broadly similar spectrum of effects on posterior axial development, indicating that individual amphibian Cdx genes have overlapping roles in posterior development. The triple knockdown gives rise to a marked increase in severity of the posterior defects. These observations argue for a model in which it is the overall level of Cdx activity in the posterior, rather the distinct function of individual Cdx genes which is required for posterior axial extension. Support for this hypothesis comes from our observation that the posterior truncation phenotype can be rescued by expression of a single mutant Cdx protein (Xcad-VP16). Furthermore, we show that double Cdx combination knockdowns can be rescued by overexpressing the third nontargeted wild-type Cdx protein. We note that rescue with the wild-type proteins is somewhat less efficient than that achieved with the mutant Cdx protein and might indicate that the wild-type Cdx proteins are subject to regulatory interactions that are not apparent with the mutant protein. Further evidence of overlapping function is provided by the additive effects of knockdown on the expression of genes in Cdx regulated pathways, such as the 5′ Hox genes, and on the development of the digestive tract, both of which are discussed in detail below.

Although we provide evidence that the amphibian Cdx proteins have similar biological activities in the processes analyzed in this study, we cannot exclude that, in a given context, individual Cdx proteins have different biological activities. Further studies involving the analysis of global changes in gene transcription, in response to knockdown of individual Cdx proteins, will be required to address this issue.

### *Cdx* Regulation of Posterior Hox Genes

There is ample evidence from both *Xenopus* and other vertebrate models that Cdx function is linked to posterior Hox gene regulation (Subramanian et al., 1995; Chawengsaksophak et al., 1997; Epstein et al., 1997; Isaacs et al., 1998; van den Akker et al., 2002). However, the relationship between Cdx levels and posterior Hox expression remains unclear. Some general principles can be drawn form the present study.

In Cdx knockdown embryos, we see effects on Hox genes from the onset of their expression (late gastrula stages) through neurula stages, indicating that Cdx function is required from early stages of development to establish and then maintain correct domains of 5′ Hox gene expression. Our data provide some evidence that knockdown of amphibian Cdx4 has somewhat stronger effects on Hox gene expression than does the single knockdown of Cdx1 or Cdx2. However, the picture that emerges is that a given Cdx gene does not regulate a subset of target 5′ Hox genes, but rather it is the overall level of Cdx activity in the embryo that is critical for normal posterior Hox gene expression. The increased effect of the triple Cdx knockdowns over the single knockdowns on Hox gene expression, and the increased effect of the double Cdx1+2 knockdown compared to the single Cdx1 or Cdx2 knockdown shown in this study also support this view. These data are in keeping with previous studies indicating that a gradient of Cdx activity in the posterior axis is required for establishing the boundaries of posterior Hox gene expression (Marom et al., 1997; van den Akker et al., 2002; Gaunt et al., 2004,^2008^).

A previous study from our laboratory used an antimorphic Cdx protein (XcadEnR) containing the DNA binding domain form Cdx4 to investigate Cdx function in *Xenopus laevis* (Isaacs et al., 1998). This mutant acts as constitutive repressor of transcription from Cdx target genes. The inhibition of posterior elongation resulting from Cdx knockdown reported here is broadly in keeping with the phenotypes that result from overexpression of XcadEnR. However, high level expression of the antimorphic protein gives rise to a much stronger phenotype of extreme posterior truncation than is seen with the knockdown strategy. This is to be expected given that overexpression of the antimorphic protein represents gain of function rather than loss of function. Similar phenotypic effects have also been reported for another antimorphic Cdx protein derived from the Cdx1 protein (Levy et al., 2002). In keeping with the present study, overexpression of XcadEnR inhibits Hox gene expression through gastrula and neurula stages and has little effect on the expression of the mesodermal marker *brachyury*.

### Other *Cdx* Regulated Pathways in the Early Embryo

We did not detect effects on the early expression of the key germ layer regulators *brachyury* or *Sox17b* in Cdx knockdown embryos. This indicates that mesoderm and endoderm are specified, although at present we cannot exclude the possibility that Cdx knockdown has other effects on the patterning of early mesodermal tissues. Similarly, we find that, despite the obvious deficit of posterior dorsal axial and paraxial tissue in later development, early markers of these tissues are unaffected by Cdx knockdown, suggesting that Cdx genes regulate other pathways than those required for the initial specification these tissues.

Canonical and noncanonical Wnt signaling pathways play key roles in the patterning and morphogenesis of the vertebrate posterior axis (Takada et al., 1994; Wallingford, 2004). Cdx regulation of these pathways provides a possible mechanism that underlies the observed Cdx knockdown phenotype of reduced axial elongation and subsequent inhibition of tail bud outgrowth. Our data support this hypothesis; we show that expression of the *Wnt3A* and *Wnt5A* ligand genes are downregulated in response to Cdx knockdown. Furthermore, we show that increasing Cdx activity upregulates expression of *Wnt3A* and *Wnt5A* in both whole embryos and tissue explants. However, our data do not indicate if the regulation of Wnt genes is direct or requires the activation of intermediate pathway components by Cdx proteins.

Wnt5a is a regulator of morphogenesis acting through a noncanonical pathway (Wallingford et al., 2001). Consistent with our findings with Cdx knockdown embryos, it has been shown that zebrafish *wnt5* (*pipetail*) mutants gastrulate normally but subsequently exhibit abnormal posterior morphogenesis (Kilian et al., 2003). It appears that, in *pipetail* mutant fish, Wnt11 is able to compensate for the lack of Wnt5 activity during gastrula stages, allowing normal gastrulation movements. Subsequently, there is increasing reliance on Wnt5 function to regulate posterior morphogenesis. In keeping with such a model, our data show that in contrast to *Wnt5a*, *Wnt11* expression is unaffected by Cdx knockdown and morphogenetic activity associated with gastrulation movements are normal. As with the *pipetail* mutant, subsequent posterior morphogenesis is inhibited by Cdx knockdown. It has also been noted that the phenotype of the *Wnt5a* null mouse is very similar to that reported for *Cdx1*^*−/−*^/^*+/−*^ mice (Yamaguchi et al., 1999; van den Akker et al., 2002).

It is possible that the reduced elongation of isolated dorsal and ventral half explants during the neurula to tail bud stage is in part due to inhibition of Wnt5a signaling. However, at present, there is no direct evidence to support a role for noncanonical Wnt signaling in regulating the cell rearrangements involved in dorsal or ventral elongation during these stages.

The canonical Wnt ligand Wnt3A is another important regulator of posterior development in vertebrates. *Wnt3a* null mice embryos lack posterior somites and fail to form a tail bud (Takada et al., 1994). In addition, Wnt3A has been implicated as a signal involved in the out growth of the tail bud during amphibian development (Beck and Slack, 1999). It has been proposed the Wnt3A is a key regulator of Cdx gene expression in the mouse and zebrafish (Shimizu et al., 2005). Our data indicate that Cdx function is itself required for normal *Wnt3A* expression in the postgastrula embryo, suggesting that Wnt3A and Cdx factors are components of a regulatory loop necessary for normal posterior development.

Wnt3A and Cdx2 knockout mice exhibit similar phenotypes (Chawengsaksophak et al., 2004). Early *Wnt3A* expression in the primitive streak is normal in *Cdx2* null mice but by the nine somite stage its expression is reduced relative to control embryos (Chawengsaksophak et al., 2004). Reduced *Wnt3A* expression at this stage was interpreted as resulting from an overall reduction in posterior development. This is unlikely to be the case in *Xenopus* because we detect reduced *Wnt3A* expression in a region of the embryo where expression of other markers such as *MyoD*, *Wnt11*, and *brachyury* are unaffected.

### *Cdx* Function in Development of the Amphibian Gut

Due to its complex, developmentally structured architecture, the vertebrate gut provides an excellent system to study the effect of factors involved in the anteroposterior patterning. In this regard, there is evidence showing that Cdx genes are involved in the regional specification of the gut. Heterozygote *Cdx2* knockout mice develop polyp-like lesions in the caecum (Chawengsaksophak et al., 1997; Beck et al., 1999). The presence of anterior-type epithelial morphology in these lesions, and the finding that ectopic *Cdx2* gene expression in the stomach results in a transformation of the gastric mucosa to a more posterior intestinal phenotype (Mutoh et al., 2002; Silberg et al., 2002), indicate that the Cdx genes act as homeotic genes to define the identity of the intestinal territory.

It has also been shown that Cdx factors are required for differentiation of the intestinal epithelium. Several studies indicate that Cdx factors directly regulate the expression from several intestine specific differentiation genes, including *sucrose isomaltase*, *MUC2*, and *KLF4* (Guo et al., 2004).

In common with other vertebrates, *Xenopus* *Cdx1* and *Cdx2* are expressed in the endoderm of the small and large intestine (Chalmers et al., 2000; Reece-Hoyes et al., 2002). We show that loss of Cdx activity in the early embryo does not alter the expression of the endodermal markers *Xsox17b*, *darmin*, and *vito*, indicating that initial endodermal specification is not affected by Cdx knockdown. Regional specification of the endoderm occurs rather late in *Xenopus* development, between tail bud and tadpole stages 25 and 35 (Horb and Slack, 2001). At these stages, our data demonstrate that regional specification is impaired in Cdx-deficient embryos. Thus, whereas the expression patterns of the endodermal markers analyzed in this study show little or no difference between early neurula and tail bud stages, from tail bud stages onward, down-regulation of posterior gut markers like *darmin* and *IFABP* indicates that regional specification is perturbed as a result of Cdx deficiency. Our results show that expression of the intestinal marker *IFABP* is almost absent in double Cdx1+Cdx2 embryos but is relatively normal in the single knockdowns, indicating that amphibian Cdx1 and Cdx2 have essential roles in specifying regional identity within the gut, and that their functions are partially overlapping. However, the loss of *IFABP* expression is not accompanied by ectopic expression of the anterior gut marker *Sox2*. Thus our experiments do not indicate that loss of Cdx function in the amphibian gut leads to posterior to anterior transformation, as has been noted in the gut epithelium of mice heterozygote for a null allele of *Cdx2* (Beck et al., 1999).

Given the *Cdx1* and *Cdx2* expression in the endoderm from tail bud stages, it seems likely that at least some of effects on regional specification in the gut in knockdown embryos at these later stages are directly due to effects on Cdx function in endodermal cells. However, a previous study indicates that the mesoderm surrounding the gut plays a key role in its regional specification (Horb and Slack, 2001). It is possible that Cdx knockdown compromises the mesoderm's role in regional specification of the gut. In this regard, we note the down-regulation of *Vent2* expression in the ventrolateral mesoderm in response to Cdx knockdown, indicating that Cdx knockdown affects the properties of the ventrolateral mesoderm involved in regional specification of the gut. The ventrally expressed mesodermal marker and hematopoietic regulator *Scl* is also down-regulated in Cdx knockdown embryos, which is in keeping with the proposed role of Cdx factors in regulating vertebrate blood formation (Davidson et al., 2003) and provides further evidence for abnormal ventrolateral mesoderm development in Cdx knockdowns.

It has been proposed that radial intercalation of endodermal cells drive the elongation and later coiling of the gut. This process occurs from stage 30 onward, accompanying cell differentiation (Horb and Slack, 2001). Our results show that the knockdown of either *Cdx1* or *Cdx2* gene function dramatically reduces the normal complex coiling of the gut, and in compound Cdx1+Cdx2 knockdown embryos the gut resembles a simple linear tube. It is interesting to speculate that the requirement for Cdx function in elongation of the posterior body axis and the elongation of the gut during gut coiling demonstrate a general role for Cdx factors in regulating tissue morphogenesis and cell movements during postneurula stages. Further studies will be necessary to determine the molecular pathways involved in regulating posterior identity and morphogenesis in the amphibian gut.

### *Cdx* Genes as Conserved Regulators of Posterior Development

There is a wealth of functional data demonstrating that a requirement for Cdx gene function in posterior axial development has been conserved during animal evolution. However, there is increasing evidence that the requirement for individual Cdx genes varies between animal groups. The evolution of the vertebrates was accompanied by an expansion in the number of Cdx genes. Thus in amphibians and amniotes three Cdx genes are present (*Cdx1*, *Cdx2*, and *Cdx4*). The orthology of the amphibian Cdx genes with the amniote Cdx genes is confirmed by analysis of their genomic context. In both amphibians and amniotes *Cdx2* is contained in the single intact ParaHox A gene cluster and *Cdx1* and *Cdx4* are contained within the degenerate ParaHox D and B clusters (Ferrier et al., 2005; Reece-Hoyes et al., 2005; Isaacs, unpublished data).

Despite the high degree of conservation in the genomic organization of the Cdx genes, important differences have emerged in how these genes are deployed during the development of amniotes and amphibians. For example, in *Xenopus tropicalis*, *Cdx4* has the most anterior boundary of expression and *Cdx1* the most posterior boundary in the dorsal axis, whereas in the mouse *Cdx1* has the most anterior boundary. This calls into question the “functional” orthology of these genes despite their proven orthology at the genomic level (Gaunt et al., 2004). We also note that outside the homeodomain regions the peptide sequences of the amphibian orthologs are relatively poorly conserved (Reece-Hoyes et al., 2002).

Analyses of single and compound Cdx knockdown in other model systems also indicate that the requirements for individual Cdx genes in posterior development vary between animal groups. Thus mutation in or knockdown of the zebrafish *Cdx4* gene leads to posterior truncation, whereas knockdown of zebrafish *Cdx1a* has no effect on posterior morphogenesis (Shimizu et al., 2005). Also, *Cdx4* knockout mice have rather normal axial development (van Nes et al., 2006), whereas *Cdx1* knockout mice have alterations in patterning of the cervical vertebrae (Subramanian et al., 1995). These relatively mild effects are in contrast with the dramatic inhibition of posterior axial development noted in *Cdx2* knockout mice (Chawengsaksophak et al., 2004). In contrast to the situation in mice and zebrafish, we find that knockdown of each of the *Xenopus* Cdx genes results in a rather similar range of effects on elongation of the posterior axis.

Despite differences in the requirement for individual Cdx genes between species, a common theme that emerges from this and previous studies is that the overall level of Cdx function in the embryo is critical in regulating posterior development. For example, the compound knockdown of zebrafish Cdx4 and Cdx1a results in synergistic reduction in posterior axial development (Shimizu et al., 2005; Davidson and Zon, 2006). Heterozygote *Cdx2* null mice exhibit mild defects in elongation of the posterior axis in comparison to the marked posterior truncation in the homozygous nulls. Similarly, axial development in *Cdx4* knockout mice is relatively normal, whereas Cdx4 knockout mice, which are also heterozygous for a *Cdx2* null allele, are truncated posterior to the hindlimbs (van Nes et al., 2006).

Evidence indicates that Cdx function is required for the regulation of similar key pathways in all vertebrate groups examined. However, there appears to be considerable variability in exactly how the individual Cdx genes are deployed in individual species to fulfill this set of conserved Cdx functions. These observations support the notion that during vertebrate evolution the main selective pressure on the complement of Cdx genes has been to maintain the overall landscape of Cdx activity in the developing embryo rather than to select for the specific functions associated with individual Cdx proteins. Relatedly, we note that the *Cdx2* gene has been lost from the teleost fish genome, while there are two Cdx1 orthologues present (Mulley et al., 2006). Such flexibility during evolution almost certainly emerges from the overlapping and partially redundant function of the three Cdx proteins. The observed variability underlines the importance of detailed analysis of Cdx function in a wide range of animal species.

## EXPERIMENTAL PROCEDURES

### Embryo Culture and Micromanipulation

*Xenopus laevis* eggs were obtained as previously described (Isaacs et al., 1992). *Xenopus tropicalis* eggs were obtained by inducing females with 100 units of human chorionic gonadotrophin (Intervet) and fertilized as previously described (Khokha et al., 2005). Staging of *Xenopus tropicalis* embryos was according to Nieuwkoop and Faber (1967).

*Xenopus laevis* embryos were injected at two- or four-cell stage with 10 or 5 nl of solution/cell, respectively (20 nl total volume/embryo). *Xenopus tropicalis* embryos were injected at two- or four-cell stage with 2.5 or 1.25 nl of solution/cell, respectively (5-nl total volume/embryo). Embryos for phenotype and in situ hybridization were injected into the marginal zone at the pigment boundary or vegetal pole region and cultured at 22°C until the desired stage. Embryos for animal cap explants were injected into the animal pole region. Animal cap explants were dissected out using sharpened tungsten needles at mid-blastula stage 8. Animal cap explants for growth factor treatment were cultured in 15-μl microtiter dish wells in 50% Normal Amphibian Medium (NAM) + 5% bovine serum albumin, either in the presence or absence of growth factors. Recombinant *Xenopus* FGF4 protein (Isaacs et al., 1992) and activin A protein (Sigma) were used at 10 U/ml and 50 U/ml, respectively.

To produce neurula stage dorsal and ventral explants, embryos previously injected with 40 ng of the Cdx1, Cdx2, and Cdx4 MO combination were dissected at stage 16 as described in Larkin and Danilchick (1999). Dorsal and ventral explants were cultured separately in MRS/3 in agarose-coated plates until stage 27. The length of explants were measured as described in Larkin and Danilchick (1999), using the Spot Junior CCD digital camera and software (Diagnostic Instruments). Statistical analysis was carried out in Excel (Student's *t*-test).

### DNA Constructs and mRNA Synthesis

The 5′ UTR and coding region of the *Cdx1*, *Cdx2*, and *Cdx4* cDNAs were polymerase chain reaction amplified using *Pfu* hi-fidelity polymerase (Stratagene) and subcloned, in frame with 6× repeats of a sequence coding for of the myc-epitope tag in the pCS2+MYC vector. Cs2+Cdx-myc constructs, Cs2+XcadVP16, Cs2+GFP, Cs2+Wnt3A and Cs2+Cdx1 were linearized with *Not*I and transcribed with the SP6 Megascript kit (Ambion) using a modified protocol including 0.5 mM GTP and 5 mM m^7^G(5′)Gppp(5′)G cap analogue (Isaacs et al., 1998).

### Morpholino Oligonucleotides (MO)

All Cdx MOs used in this study were targeted to the 5′UTR region of the gene and/or the start site of translation. The set-1 antisense MOs were kindly provided by E. Amaya. The set-2 antisense MOs, set-1 five mismatch Cdx MOs, and the standard control MO were obtained from GeneTools, LLC. The sequences of the MOs used in this study are as follows: *Standard Control*, 5′ CCTCCTACCTCAGTTACAATTTATA 3′. *Set 1 MOs:* Cdx1/Xtcad2, 5′ CGGGTAACAATCTCCTGAGTCTGTG 3′; Cdx2/Xtcad1, 5′ AACAAGTAACTCACGTACATGGCGG 3′; Cdx4/Xtcad3, 5′ ATCCTTGGTGGTCATCTTTATCCTC 3′; Cdx1 mismatch control, 5′ CGcGTAAgAATCTgCTGAcTCTcTG 3′; Cdx2 mismatch control, 5′ AAgAAcTAACTgACGTAgATGcCGG 3′; Cdx4 mismatch control, 5′ ATgCTTcGTGcTCATgTTTATgCTC 3′. *Set 2 MOs:* Cdx1/Xtcad2, 5″ ATCCAAAAGATAACCCACGTACATC 3″; Cdx2/Xtcad1, 5′ CTCCAACAAGTAACTCACGTACATG 3′; Cdx4/Xtcad3, 5′ CTAGGCGAGATCCTTGGTGGTCATC 3′. Lower case letters indicate changes in bases respect to the corresponding antisense MO.

### Western Blotting

Embryos were harvested at late blastula stage 9, homogenized in lysis buffer (0.2 M sucrose, 100 mM Tris pH 8, 10 mM CaCl_2_, 10 mM Mg_2_Cl, 4 μl/embryo) on ice and centrifuged for 10 min at 4°C. Protein concentration in the supernatant was determined using the BioRad colorimetric assay (Bio-Rad, Hercules, CA). Equal protein amounts per sample were analyzed on 7.5% sodium dodecyl sulfate-polyacrylamide gel electrophoresis, transferred onto Immobilon-P membranes (Millipore, Billerica, MA) and subsequently probed with mouse anti-myc antibody (9B11, 1:5,000, Cell Signalling Technology, Inc.) overnight at 4°C. After extensive washing, membranes were probed with rabbit anti-mouse secondary antibody conjugated with peroxidase (1:2,000; Amersham) for 1 hr at room temperature, and developed using the ECL Chemoluminescence kit (Roche Diagnostics). Glyceraldehyde-3-phosphate dehydrogenase (GAPDH; 1:2,000, Insight Biotechnologies, Inc.) was used as loading control.

### Histology

Embryos were fixed in 4% paraformaldehyde /phosphate buffered saline, stained in borax carmine, dehydrated through an ethanol series and embedded in Paraplast (Sigma). Microtome sections were counterstained with picroblue-black and mounted in Histomount (National Diagnostics). Specimens were photographed using a Spot Insight CCD digital camera (Diagnostics Instruments) and image manipulation was carried out using Adobe Photoshop.

### Whole-Mount In Situ Hybridization

Antisense digoxigenin (DIG) -labeled probes were synthesized using 10× DIG labeling mix (Roche Diagnostics). Templates were linearized and transcribed as follows: *X. laevis* cDNAs *HoxD9* and *HoxD10* *Nco*I/SP6; *MyoD*, *Xbra*, and *SCL* *Xho*I/T7, *Wnt3A* BamHI/T3, *Wnt5A* BstXI/SP6, IFABP *Xho*I/T7, *Xsox2* EcoRI/T7, and *X. tropicalis* cDNAs *Cdx2* (*Xtcad1*) NdeI/T7, *Cdx1* (*Xtcad2*) EcoRV/T3, *Cdx4* (*Xtcad3*) PvuII/T7; *Wnt11* DraI/T7; *HoxA9*, *HoxA11*, and *HoxC11* SpeI/T7; *HoxC8* and *HoxC9* SacII/SP6, *HoxB9*, *Xhox3*, *HoxA7* *darmin*, *vito*, *vent-2*, and *Sox17b* EcoRI/T7. Whole-mount in situ hybridization was performed as described in Harland (1991), with minor modifications (Reece-Hoyes et al., 2002). Proteinase K treatment was carried out for 10 to 25 min with 10 μg/ml of proteinase K (Roche Diagnostics). Specimens were photographed using a Spot Junior CCD digital camera (Diagnostic Instruments), and image manipulation was carried out using Adobe Photoshop Elements.
