# Supplementary figures and images for: Overlapping functions of Cdx1, Cdx2, and Cdx4 in the development of the amphibian Xenopus tropicalis
Source: Dev Dyn. 2009 Apr;238(4):835–52. doi: 10.1002/dvdy.21901 (PMC2701559; doi:10.1002/dvdy.21901)

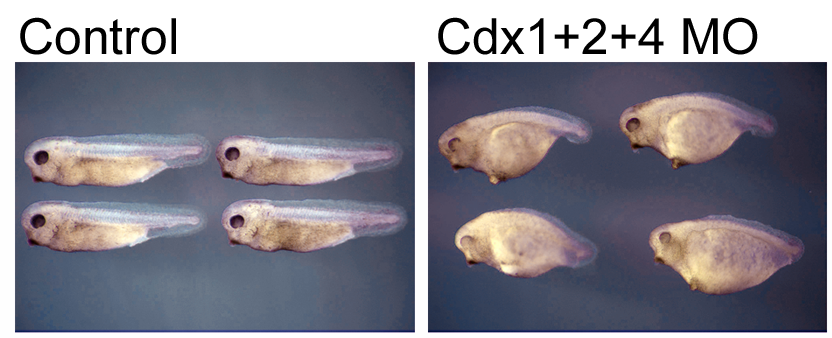

Supplement: Supplementary file 1 [file dvdy0238-0835-SD1.tif]
